# Characterization of the glycerol-3-phosphate acyltransferase gene and its real-time expression under cold stress in *Paeonia lactiflora* Pall

**DOI:** 10.1371/journal.pone.0202168

**Published:** 2018-08-10

**Authors:** Xueting Li, Ping Liu, Panpan Yang, Chuanzhu Fan, Xiaomei Sun

**Affiliations:** 1 Horticulture College, Shenyang Agricultural University, Shenyang, Liaoning, China; 2 Forestry College, Shenyang Agricultural University, Shenyang, Liaoning, China; 3 Department of Biological Sciences, Wayne State University, Detroit, MI, United States of America; University of Arizona, UNITED STATES

## Abstract

Elucidating the cold tolerance mechanism of *Paeonia lactiflora*, which is one of the most valuable ornamental and medicinal plants in Asia, fundamentally impacts its breeding and production. The glycerol-3-phosphate acyltransferase (GPAT) gene plays a pivotal role in cold resistance in a variety of plant species. Here, we cloned the *P*. *lactiflora GPAT* gene, determined its expression pattern, and tested its role in cold resistance. We obtained the full-length *P*. *lactiflora GPAT* gene using tissue-cultured seedlings and real-time polymerase chain reaction and rapid amplification of cDNA ends analyses. We named this gene *PlGPAT* in *P*. *lactiflora*. Phylogenetic analysis indicates that the *PlGPAT* gene is closely related with the *GPAT* genes in core eudicots. The phylogenetic tree containing 31 angiosperm species based on *GPAT* protein sequences is largely consistent with the known phylogeny in flowering plants. We conducted a time-course *PlGPAT* expression analysis and demonstrated that *PlGPAT* expression is correlated with low-temperature stress. Our results suggest that the *PlGPAT* gene plays an important role in regulating cold resistance in *P*. *lactiflora*.

## Introduction

*Paeonia*, the only genus in the family Paeoniaceae, contains some of the most prestigious and valuable ornamental flowering plants. The current *Paeonia* genus contains 33 known species [1]. *Paeonia* species are mostly distributed in Asia, Europe and Western North America. In general, *Paeonia* plants have high ornamental, medicinal, and economical value [2]. Within *Paeonia*, *Paeonia lactiflora* Pall (Chinese peony or common garden peony) is an herbaceous perennial plant and is one of the most important traditional flowering plant species in China. *Paeonia lactiflora* is a typical temperate zone plant that can adapt to a wide range of ecological conditions. More importantly, *P*. *lactiflora* is highly cold-tolerant, making it an excellent genetic resource for cold resistance breeding [3]. However, studies focused on the cold hardiness of *P*. *lactiflora* are relatively limited, and previous studies have mostly focused on the physiological changes in *P*. *lactiflora* during cold treatment [4,5]. Furthermore, the *P*. *lactiflora* cold resistance gene has not been identified or cloned. Therefore, the molecular mechanism for cold hardiness in *P*. *lactiflora* remains to be explored.

Cell membrane fluidity plays an important role in maintaining biological metabolic activities in flowering plants. Low temperature can decrease the biological cell membrane fluidity and increase protein activation in the membrane. As a result, cold conditions alter the state of the biofilm from a liquid crystalline state to a crystalline solid state. Moreover, under frigid conditions, the biofilm’s function in active transportation and selective permeability is reduced or even lost, which is very harmful to plants [6]. Under cold stress, lower membrane fluidity in cells helps to maintain membrane proteins and enzyme transcription in the fatty acid synthesis pathway [7]. The degree of unsaturation in lipoids and fatty acids in membrane lipids greatly influences their phase-transition temperature. Higher degrees of lipid unsaturation can result in lower phase-transition temperatures, which leads to increased cold tolerance in plants [8,9]. Therefore, the lower the plant phase-transition temperature is, the better the cold tolerance of the plant will be. It is known that phosphatidylglycerol (PG) *cis*-unsaturated fatty acid levels directly determine the fluidity of the phospholipid bilayer. Previous research showed that the cold resistance ability of a plant is closely related to the phosphatidylglycerol levels of *cis*-unsaturated fatty acids in chloroplast membrane lipids. The PG sn-1 position in cold resistance plants had a higher proportion of *cis*-unsaturated fatty acid, while it had lower levels in cold-sensitive plants [10, 11]. Glycerol-3-phosphate acyltransferase (GPAT) is the first acyl esterase in the phosphatidylglycerol biosynthesis of biofilms [12, 13]. Cold-resistant plants preferably have C18:1-ACP as the substrate, and GPAT converts the fatty acyl group at the C-1 position into 3-phosphoglycerol to synthesize 1-acyl-S-glycerol-3-phosphate. After being catalyzed by acyl fatty acid desaturase, 1-acyl-S-glycerol-3-phosphate continues desaturation to increase the level of *cis*-unsaturated fatty acids in phosphatidylglycerols. Therefore, the ability of plants to resist the cold is improved through this process [10, 14].

Cold tolerance genes can be divided into two major categories: regulatory genes and functional genes. When the plant is subjected to low-temperature stress, the cold regulatory genes, such as *COR* (cold-regulated), *CRP* (CRT/DRE binding factor), and *ICE* (inducer of CBF expression), regulate the expression of functional cold-resistant genes through signal transduction. Functional cold-resistant genes such as *AFP* (antifreeze protein), *SOD* (superoxide dismutase), *CAT* (catalase), *POD* (peroxidase), *FAD* (fatty acid desaturase), and *GPAT* directly protect cells from hypothermic damage by encoding proteins. These encoded proteins improve plant cold resistance [15]. It is widely believed that glycerol-3-phosphate acyltransferases play an important role in the cold-resistant ability of plants. The effect of the *GPAT* gene on plant cold hardiness has been confirmed to be significant. For example, introducing the *GPAT* gene from cold-sensitive pumpkin into tobacco increased the saturation degree of fatty acids in transgenic tobacco membrane lipids. After introducing the *GPAT* gene from cold-tolerant *Arabidopsis thaliana* into tobacco, the fatty acid composition of thylakoid PG in the transgenic plants tended to be unsaturated. This resulted in greatly increased cold tolerance in the transgenic plants [8]. Yokoi et al. (1998) introduced the *GPAT* gene from *Arabidopsis thaliana* into rice, which increased the unsaturated fatty acid contents in PG and enhanced the cold-resistant ability of rice [16]. Ariizumi et al. (2002) transferred *GPAT* genes from spinach and *A*. *thaliana* into rice, which increased the *cis*-unsaturated fatty acids in PG from 19.3% to 32.0% and 29.4%, respectively. In rice leaves, the cold-resistant ability of transgenic rice also correspondingly improved [17]. Since the ability to resist the cold is very important to plants, especially in colder regions around the globe, studying *GPAT* gene function in a variety of plants requires exploration.

To elucidate the function of this gene in plants, it is necessary to obtain full-length DNA sequences or cDNA sequences [18]. Some *GPAT* gene cDNA or DNA fragments have already been cloned from many plants [19–28]. However, the characterization of the *GPAT* gene in *Paeonia lactiflora* has not yet been reported. Here, we isolated, cloned, and sequenced the *P*. *lactiflora GPAT* gene and its full-length transcript. We further determined its physicochemical characteristics and biophysical features (e.g., structural domain, transmembrane and signal sequence status, and secondary and spatial structures), as well as the phylogenetic relationships among *GPAT* genes in flowering plants. More importantly, we performed transcription analysis in a cold-induced environment (different treatment times) using real-time quantitative polymerase chain reaction (RT-qPCR) to infer the biological functions of the *GPAT* gene during low-temperature stress resistance in *P*. *lactiflora*. Our results provide fundamental data that reveal cold resistance mechanisms, which can be applied to improve cold resistance in target plants using transgenic technologies.

## Materials and methods

### Plant materials and treatments

Underground buds of *P*. *lactiflora* ‘Fen Yu Nu’ were used as tissue culture explants to obtain plantlets using previously described culture methods [29]. Well-grown plantlets were selected for gene cloning. The plantlets were treated at 4°C for seven different time points, including 2, 4, 8, 12, 24, 48, and 72 hours. Tissue-cultured seedlings that were not exposed to cold treatment were used as a positive control and marked as 0 h. Each sample had three replicates. The leaves from the tissue-cultured seedlings were cut and wrapped in aluminum foil. All samples were frozen immediately in liquid nitrogen and stored at −80°C until further analyses.

### RNA extraction and cDNA amplification of the *GPAT* gene

Total RNA was extracted from *P*. *lactiflora* tissue-cultured seedlings using the RNAprep pure Plant Kit (Tiangen, Beijing, China) according to the manufacturer’s instructions. First-strand cDNA was synthesized from total RNA using the Oligo d(T)_15_ primer and M-MLV reverse transcriptase (Promega, Shanghai, China) according to the manufacturer’s instructions.

All primers used in this study were designed with Primer Premier 5.0 [30] and are listed in Table 1. The first-strand cDNAs were used as the template, and the conserved *P*. *lactiflora GPAT* cDNA fragment was amplified with the GF1, GR1, GF2, and GR2 degenerate primers (Table 1). Two rounds of PCR were conducted with the following conditions: (1) 94°C for 5 min; (2) 35 cycles of 94°C for 30 s, 59°C for 30 s, and 72°C for 1 min; and (3) 72°C for 10 min.

**Table 1:** Primers used in this study.

Primer	Primer sequence(5′→3′)	Procedure
GF1	5′-ATATTGC(T/A)G(A/C)AGGAATGGA(G/A)GA-3′	EST isolation
GR1	5′-GGAGG(G/A)GGCAT(A/G)AT(A/G)TCAT-3′	EST isolation
GF2	5′-AATGGA(G/A)GA(A/G)(C/A)TGTAT(C/T)AGAA(T/C)TA-3′	EST isolation
GR2	5′-G(A/C)CCAGG(G/A)ACACCAG(C/A)ATGTT-3′	EST isolation
GPAT3-1	5′-GTATTTTCTTGGAAGTTGAGGACCC-3′	3′RACE
GPAT3-2	5′- CAATGCGAGAGCCTTTTGACTACTA-3′	3′RACE
GPAT3-3	5′-TTGTTACTTGAAAAAACGAATCCAC-3′	3′RACE
AP	5′-GGCCACGCGTCGACTAGTACTTTTTTTTTTTTTTTTT-3′	3′RACE
AUAP	5′-GGCCACGCGTCGACTAGTAC-3′	3′/5′RACE
GPAT5-1	5′-GTAGTCAAAAGGCTCTCGCATTGCTTTG-3′	5′RACE
GPAT5-2	5′-CTGCCCTGGGATCTCCACTTTGG-3′	5′RACE
AAP	5′-GGCCACGCGTCGACTAGTACGGGGGGGGGGGGGG-3′	5′RACE
GPATF	5′-ACTATGTTGCTATTCTCTTCTTCGC-3′	ORF subclone
GPATR	5′-GAAGGTTGACTAAACCCACGGTT-3′	ORF subclone
ACTINF	5′-GGTCTATTCTTGCTTCCCTC-3′	RT-qPCR
ACTINR	5′-CCCTCTGCGTCTACACTTTC-3′	RT-qPCR
F	5′-AAATTGGGGAAAAGCGAGTGA-3′	RT-qPCR
R	5′-GACAAAGAGACGGCTGGAGTA-3′	RT-qPCR

Based on the sequences of the conserved cDNA fragments, we designed specific primers for three genes, including *GPAT3-1*, *GPAT3-2*, and *GPAT3-3* (Table 1). A 3′-rapid amplification of cDNA ends (RACE) analysis was conducted using three sequential rounds of nested PCR. Each round of PCR performed amplification with specific and AUAP universal primers (Table 1). The following PCR cycling conditions were used for all the reactions: 94°C for 5 min 10 cycles of 94°C for 30 s, 65°C (−2°C/every two cycles) for 45 s, and 72°C for 1 min; 22 cycles of 94°C for 30 s, 55°C for 45 s, and 72°C for 1 min; and a final extension at 72°C for 10 min.

The specific 5′-RACE *GPAT5-1* and *GPAT5-2* primers were also designed based on the conserved *GPAT* gene cDNA fragment sequence (Table 1). Amplification for 5′-RACE was performed with LA Taq DNA polymerase (TaKaRa Bio, Shiga, Japan) and two rounds of nested PCR. The amplification conditions were as follows: 94°C for 5 min; 35 cycles of 94°C for 30 s, 60°C for 45 s, and 72°C for 90 s; and a final extension at 72°C for 10 min.

All PCR products were separated on 1.0% agarose gels and purified with the Quick Gel Extraction Kit (Kangwei, Beijing, China). The purified PCR products were ligated into the pGM-T vector (Tiangen) and transformed into *E*. *coli* DH5α competent cells (TaKaRa Bio, Shiga, Japan). The recombinant colonies were identified through blue-white color selection. Plasmids for positive clones were extracted using the TIANprep Mini Plasmid Kit (Tiangen, Beijing, China) and were sequenced at Shanghai Sangon Biological Engineering Technology & Services Co., Ltd. (Shanghai, China).

The conserved cDNA fragment and the 3′- and 5′-terminal sequences were assembled using the DNAMAN software (Lynnon LLC., San Ramon, CA, USA) to obtain the hypothetical full-length cDNA sequences of the *P*. *lactiflora GPAT* gene. The specific GPATF and GPATR primers (Table 1) were designed according to the open reading frame (ORF) sequence after a preliminary analysis of the full-length cDNA gene sequences. Full-length cDNA was amplified using LA Taq DNA polymerase and the following conditions: 94°C for 5 min; 35 cycles of 94°C for 30 s, 61°C for 45 s, and 72°C for 2 min; and 72°C for 10 min. The PCR product was cloned and sequenced to verify the *P*. *lactiflora* GPAT ORF sequence.

### Sequence analysis and phylogenetic tree construction

A sequence analysis was performed using Blast software (http://blast.ncbi.nlm.nih.gov/). The physicochemical characteristics of the GPAT protein were analyzed using the ProtParam tool (http://www.expasy.ch/tools/protparam.html). The amino acid sequences of homologous *GPAT* genes from 31 angiosperm species were retrieved from GenBank (S1 File). The amino acid sequences were aligned with Muscle [31]. Phylogenetic analyses were performed based on the maximum likelihood (ML) method, and the model test was used to determine the best-fit model for the ML analysis. We chose the ML method for phylogenetic tree construction, as this algorithm has clear benefits over distance or parsimony methods for analyzing the sequence evolution process [32]. A bootstrap analysis with 2000 replicates was performed simultaneously for all analyses to obtain the statistical support for each internal and external branch. All analyses were implemented in MEGA ver. 7.0 [33].

The conserved GPAT structural domain was predicted using the National Center for Biotechnology Information (NCBI) conserved domain database (CDD) at the NCBI website (http://www.ncbi.nlm.nih.gov/Structure/cdd/wrpsb.cgi) [34]. The amino acid sequence was subjected to the TMHMM server (http://www.cbs.dtu.dk/services/TMHMM/) for the transmembrane analysis, and the SignalP 3.0 Server (http://www.cbs.dtu.dk/services/SignalP/) was used to predict the protein signal sequence [35].

### Real-time quantitative analysis of *GPAT* gene expression

*Paeonia lactiflora GPAT* gene expression levels under low temperature (4°C) were measured by RT-qPCR using the SYBR® Select Master Mix (Takara Bio Inc., Japan). Specific primers (PlGPATF and PlGPATR; Table 1) were designed based on the regions of sequence alignment near the 3′-ends. First-strand cDNA synthesis was performed by reverse transcription with DNase I-treated total RNA. The cDNA fragments were amplified using the PlGPATF and PlGPATR primers targeting a 223-bp *GPAT* fragment. The *β-actin* housekeeping gene was used as an internal control to adjust for different quantities of cDNA [36]. A 218-bp β-actin fragment was amplified using the ACTINF and ACTINR primers (Table 1) based on *P*. *lactiflora β-actin* gene (GenBank accession no. JN105299.1). PCR-grade water replaced the cDNA template as a negative control.

The RT-qPCR assay was performed on a LightCycler 480 instrument (Roche, Manheim, Germany). The amplifications were performed in a 20 μL reaction volume containing 2 μL of cDNA, and the following program was used for these reactions: 95°C for 2 min; followed by 40 cycles of 95°C for 15 s, 60°C for 30 s, and 72°C for 30 s (plate read); and a final step at 72°C for 3 min. After the program finished, the C_t_ values of the *GPAT* and *β-actin* genes were obtained for each sample. The C_t_ value is defined as the PCR cycle number where the fluorescence signal crossed a threshold line placed in the exponential phase of the amplification curve. The relative expression level of the gene was calculated using the 2^−ΔΔCt^ method [37]. The *GPAT* gene expression levels of the control group were used as a calibrator and set to 1.0. The statistical analysis was performed using EXCEL (Microsoft, Inc. Redmond, WA, USA).

## Results

### Isolation of cDNA from the *P*. *lactiflora GPAT* gene

**Fig 1:**
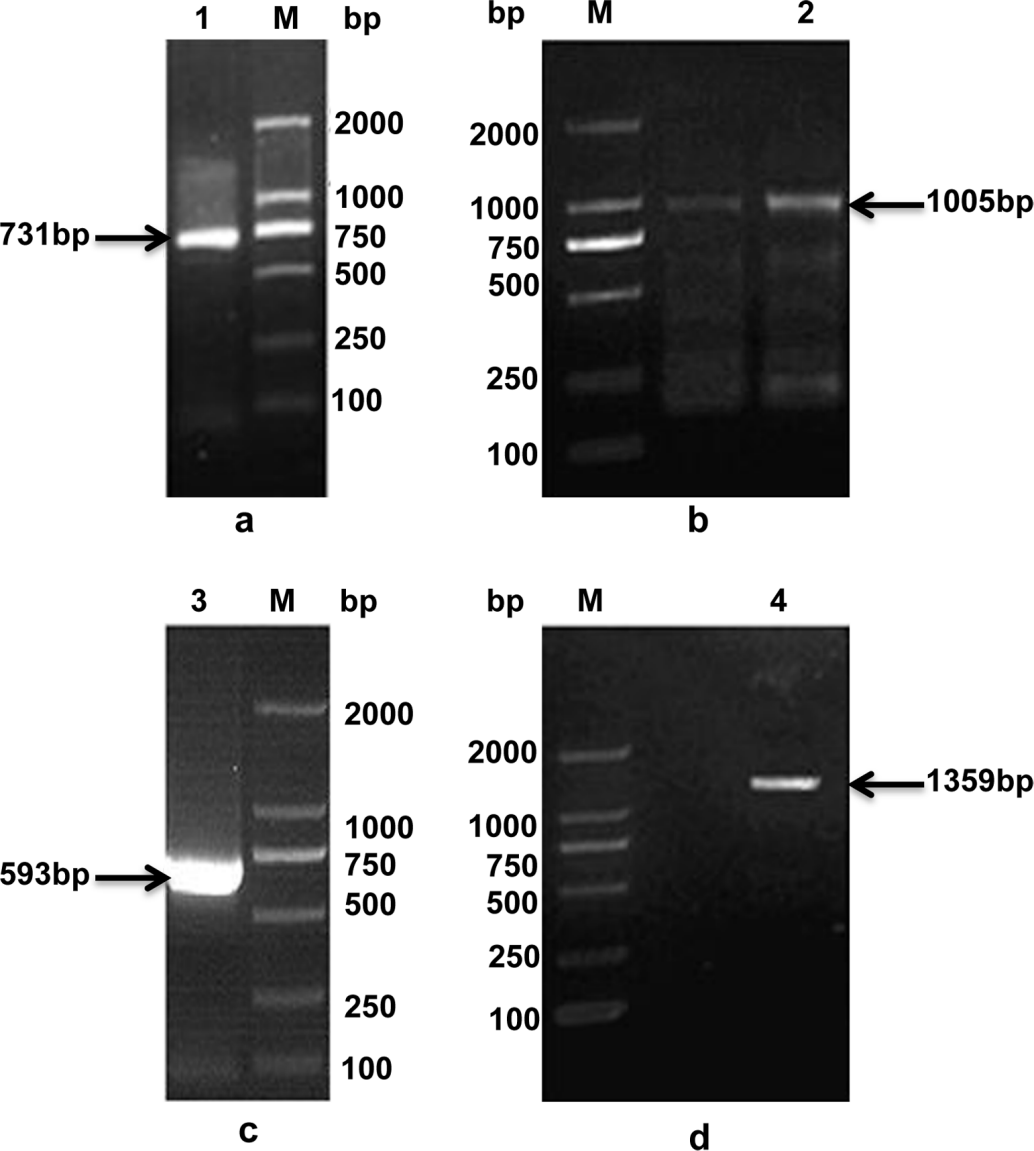
Isolating the glycerol-3-phosphate acyltransferase (*GPAT*) gene fragments. (a) The expressed sequence tag (EST) is a 731 bp fragment. M: DNA marker; lane 1: Polymerase chain reaction (PCR) product. (b) 3′-Rapid amplification of cDNA ends (RACE) 1,005 bp fragment. M: DNA marker; lane 2: PCR product. (c) 5′-RACE 593 bp fragment. M: DNA marker; lane 3: PCR product. (d) The 1,359 bp fragment including the open reading frame (ORF). M: DNA marker; lane 4: PCR product.

Using total RNA from *P*. *lactiflora* tissues as the reverse transcription template, we amplified, cloned, and sequenced a DNA fragment with an expected length of 731 bp using the GF2 and GR2 primers (Fig 1). Based on the conserved sequence of this cDNA fragment, we performed a 3′-RACE reaction and obtained the 1,005 bp 3′-end fragment (Fig 1). Furthermore, two 5′-RACE reactions were implemented, resulting in two 593 bp DNA fragments at the 5′-end (Fig 1). We also obtained a 1,359 bp cDNA when verifying the *P*. *lactiflora GPAT* gene ORF sequence with 3 bp in the 5′- untranslated region (UTR), 12 bp in the 3′-UTR and 1,347 bp in the complete ORF based on sequence assembly and a preliminary analysis (Fig 1).

### Structure and function of *P*. *lactiflora GPAT*

**Fig 2:**
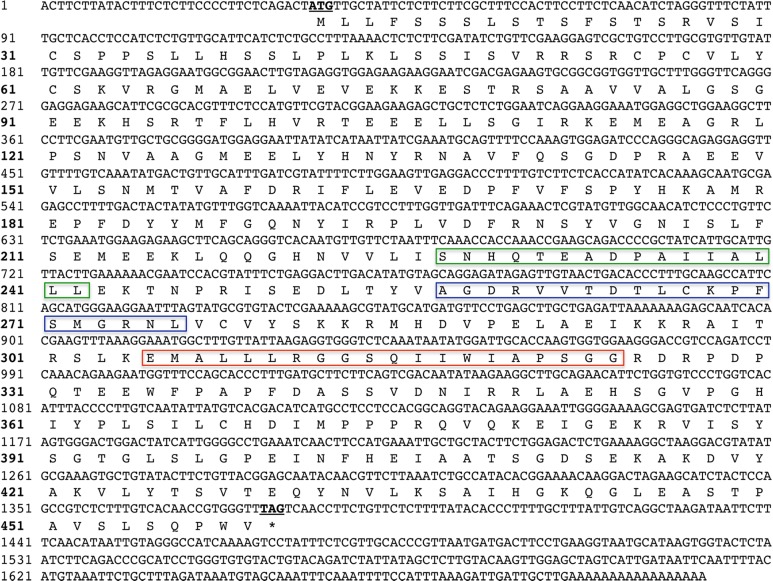
Full-length *Paeonia lactiflora* glycerol-3-phosphate acyltransferase gene *(PlGPAT)* nucleotide and translated amino acid sequences. The amino acid sequences are indicated by the single letter code. The potential translation initiation codon (ATG) is underlined and the termination codon is marked with an asterisk. The different color boxes represent three regions that are relatively conserved when comparing *PlGPAT* to homologous sequences in other plants.

The analysis of the nucleotide sequence showed that the *P*. *lactiflora GPAT* gene transcript contains 1,702 bp, with a 1,347 bp ORF, a 33 bp 5′-UTR, and a 322 bp 3′-UTR. The *P*. *lactiflora GPAT* ORF encoded a 448 amino acid protein (Fig 2). ProtParam tool analysis revealed that the molecular weight of this peptide was 49.997 kDa and the isoelectric point was 6.52. We named this *P*. *lactiflora GPAT* gene “*PlGPAT”* (GenBank accession number: KJ914575).

A complete homologous BLAST search demonstrated that the *PlGPAT* amino acid sequence was highly similar (60–75%) to other flowering plant *GPAT* genes. This indicated that we have successfully cloned the *P*. *lactiflora GPAT* gene. The amino acid sequences had the highest shared traits (75%) with the published *Vitis vinifera GPAT* (XP_002276101.1) and *Theobroma cacao GPAT* (XP_007040713.1). The amino acid sequence shared 67% sequence similarity with *Arabidopsis thaliana GPAT* (BAA00575.1).

**Fig 3:**
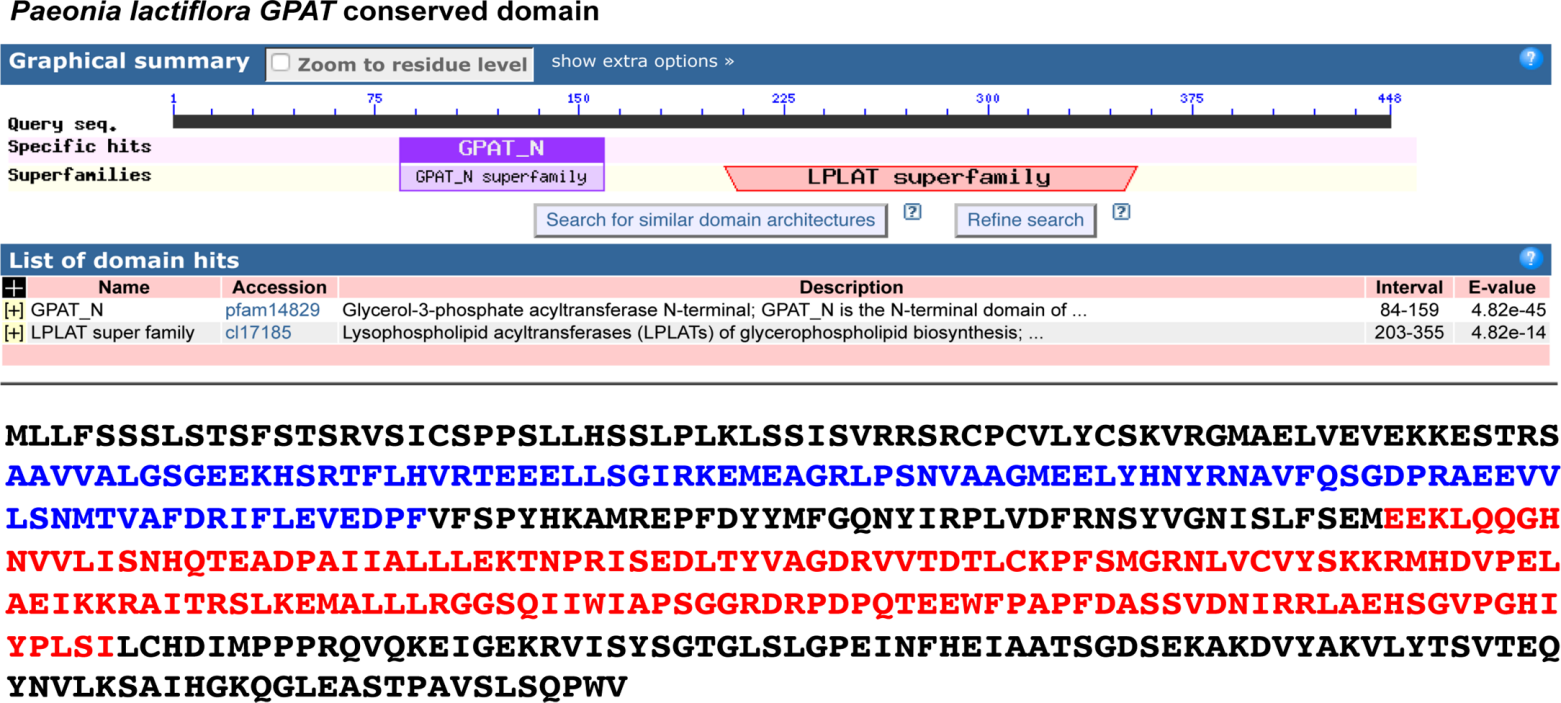
Conserved domains in *PlGPAT* detected using the NCBI Conserved Domains Search. The deduced 448-amino-acid sequence was used for the search. Two conserved regions including the GPAT_N domain (protein sequence in the blue box) and the LPLA T_GPAT domain, as well as the PLN02349 (protein sequence in the red box), are predicted.

Furthermore, we employed the CDD from NCBI to predict the PlGPAT structural and functional domains (Fig 3). The putative PlGPAT polypeptide contains two conserved regions, including the GPAT_N domain and the LPLAT_GPAT domain (Fig 3). LPLAT_GPAT is located between amino acids 189 and 424 and belongs to the lysophospholipid acyltransferase superfamily, which includes GPAT. Thus, the LP LAT_GPAT domain plays a major role in the GPAT enzyme. A Eukaryotic Neural Network search using SignalP 3.0 demonstrated that this polypeptide had no signal peptide. This polypeptide is also predicted to be a nonsecretory protein by Markov models (HMM) in SignalP 3.0. The TMHMM Server v. 2.0 predicts that this GPAT protein has no transmembrane domain.

### Phylogenetic analysis of GPAT amino acid sequences

**Fig 4:**
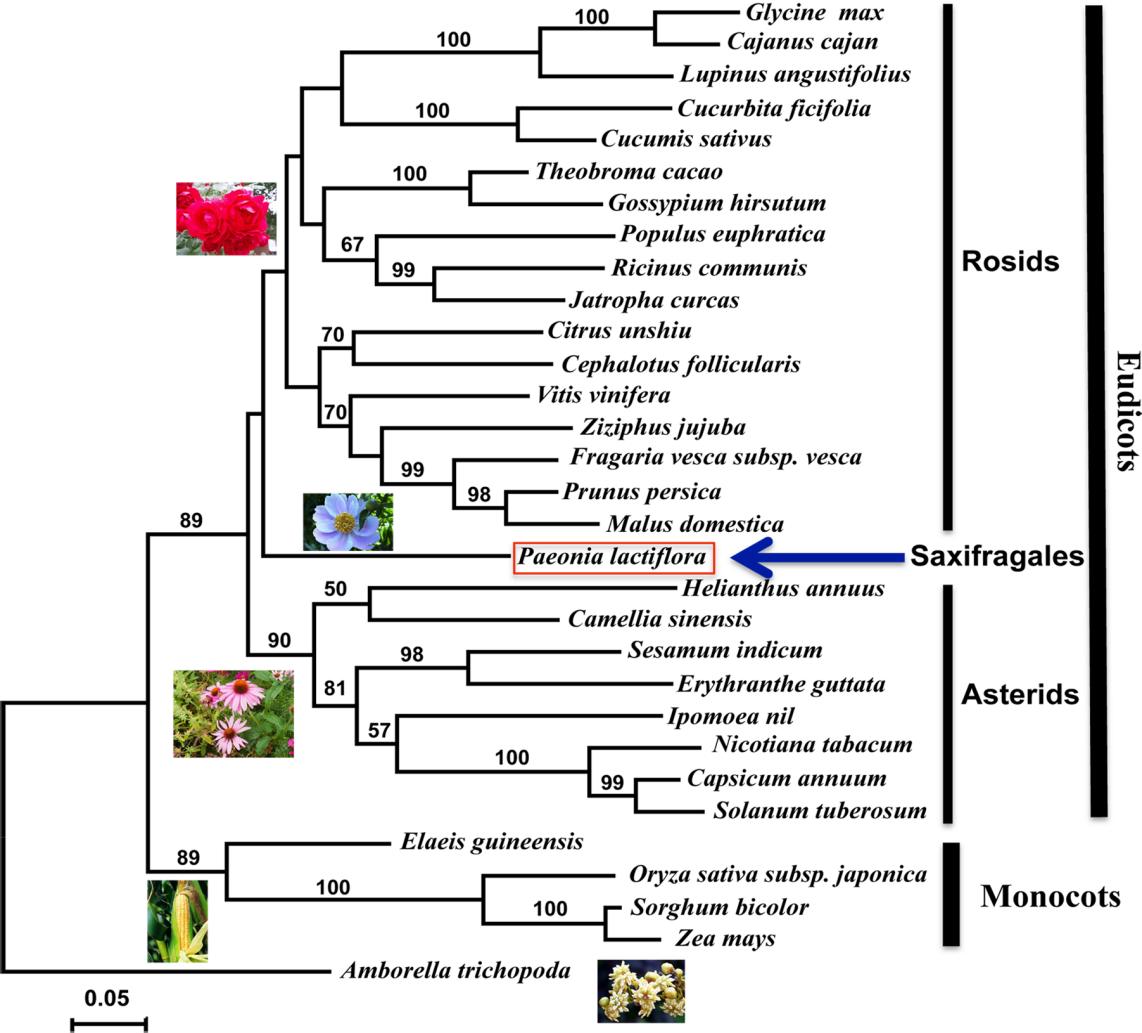
Molecular phylogenetic tree using the maximum likelihood method for the *GPAT* gene from 31 flowering plant species. The evolutionary phylogram tree was inferred using the Maximum Likelihood method based on the General Reverse Transcriptase model. The tree with the highest log likelihood (-8282.0517) is shown. Initial tree(s) for the heuristic search were obtained automatically by applying the Neighbor-Joining and BioNJ algorithms with a matrix of pairwise distances estimated using the JTT model, and then selecting the topology with a superior log-likelihood value. A discrete Gamma distribution was used to model the evolutionary rate differences among sites (5 categories (+G, parameter = 1.2311)). The rate variation model allowed for some sites to be evolutionarily invariable ([+I], 19.4199% sites). The tree is drawn to scale, with branch lengths measured as the number of substitutions per site. The tree was rooted using *Amborella trichopoda* as an outgroup. Bootstrap supports (%) with >50% are shown above branches.

The results from the phylogenetic analysis using GPAT amino acid sequences from a broad range of flowering plant species indicated that the topology of the *GPAT* gene tree is largely consistent with the species tree (Fig 4). Using *Amborella trichopoda*, the basal angiosperm species as the outgroup, eudicots and monocotyledon clearly form monophyletic groups with high bootstrap support. Two major clades are formed within the eudicot group, including Rosids containing 17 species and Asterids containing 8 species. However, this analysis is not supported by the statistical bootstrap values (Fig 4). *Paeonia lactiflora*, a species from Saxifragales, has a sister relationship with Rosids, although this analysis is also not supported by statistical bootstrap values (Fig 4). Phylogenetic analysis using *GPAT* genes from flowering plants clearly indicate the consistent evolutionary pattern and sequence evolution of *GPAT* genes, which can be used to infer the phylogeny of high hieratical angiosperm species.

### *GPAT* expression pattern under cold treatment

The expression pattern of *P*. *lactiflora PlGPAT* was analyzed at different times under 4°C using both conventional PCR and real-time quantitative PCR (RT-qPCR). We first tested the specificity of the primers for the positive control *β-actin* (ACTINF and ACTINR) and the *PlGPAT* gene (F, R). These results showed that the products amplified by conventional PCR had no primer dimers. The melting curve showed that both the *β-actin* and *PlGPAT* primers have only one T_m_ value, at 83°C and 81°C, respectively. These results indicate that both primer pairs are highly specific, and their fluorescence curves accurately reflect the amplification of the desired products.

**Fig 5:**
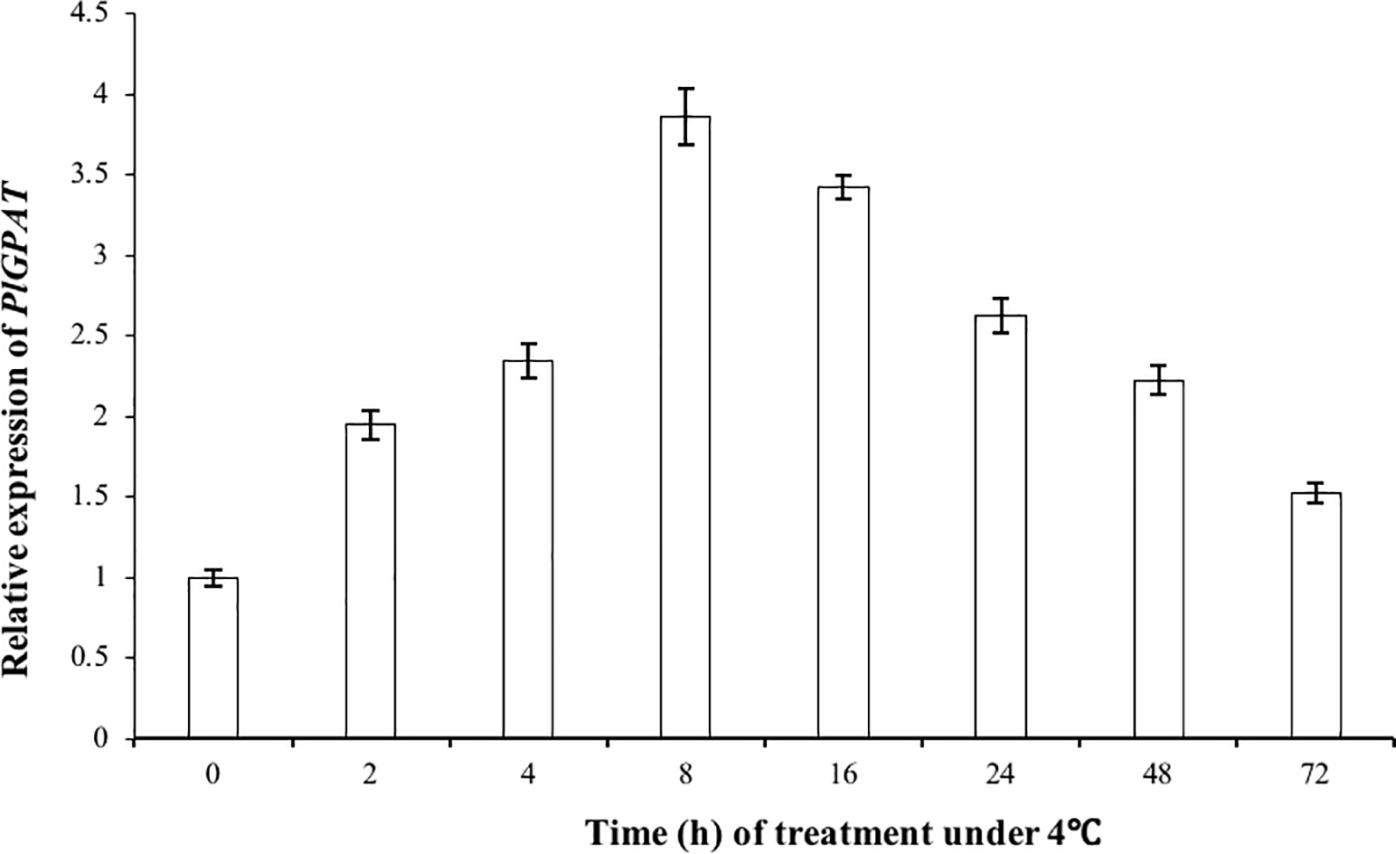
Relative *PlGPAT* gene expression levels at different time points under 4°C treatment. Transcripts for all samples were assessed by real-time quantitative polymerase chain reaction. Relative *GPAT* gene expression is shown as the ratio of *GPAT* mRNA/β-actin mRNA normalized to the *GPAT* gene expression level in the control group.

*PlGPAT* gene expression profiles are plotted in Fig 5. *PlGPAT* expression gradually increased during the 72 hours of low-temperature treatment and reached peak expression after 8 hours, resulting in a four-fold change compared to the control (0 h). Subsequently, the expression level dropped but still maintained at a relatively high level even after 72 h treatment (Fig 5).

## Discussion

The stability and mobility of the cell membrane is an important factor that affects the tolerance of flowering plants to cold temperatures. There is a close correlation between plasma membrane fatty acid unsaturation and cold sensitivity in higher plants. As previously discussed, GPAT is a key enzyme in the fatty acid desaturase metabolic pathway [38]. Many studies have shown that *GPAT* genes are closely related to cold hardiness in plants. *Paeonia lactiflora* is an important ornamental, cold-resistant, and medicinal plant species. After we first cloned the *GPAT* gene, the preliminary analysis indicated that it may have a critical function in the process of cold stress. The sequence homology and phylogenetic analyses indicate that *PlGPAT* is closely related to *GPAT* genes from core eudicots.

When the *P*. *lactiflora GPAT* gene is compared with other amino acid sequences from other plants, *GPAT* genes across different plants are relatively conserved, especially in the three main regions (Fig 3). We analyzed these conserved regions and found that the histidine (H) and aspartic acid (D) amino acid residues in the first conserved region and a glycine (G) amino acid residue in the third conservative region are highly conserved. These amino acid residues are considered to be important catalytic sites in the acyltransferase family. Additionally, phenylalanine (F) and arginine (R) in the second conserved region and serine (S) in the third conserved region play an important role in the binding of the glycerol-3-phosphate acyltransferase substrate. Moreover, further analysis reveals that approximately 100 amino acids in the upstream GPAT protein have low homology, which may be related to the acyltransferase’s different functions across various plants.

*PlGPAT* encodes a protein predicted to be hydrophilic with two conserved domains but without a transmembrane domain or signal peptide sites. Among the two conserved domains, the GPAT_N protein domain is responsible for catalyzing glycerol-3-phosphate sn-1 hydroxyl acylation, the initial step in glycerolipid synthesis. LPLAT_GPAT belongs to the lysophospholipid acyltransferase superfamily. Most enzymes in this super family are acyltransferases that catalyze reactions between acyl groups, such as acylCoAs or acylACPs, and receptor proteins [39]. Thus, the LPLAT_GPAT domain is the main conserved domain in *PlGPAT* that plays a major role in the GPAT enzyme. These structural characteristics of the *GPAT* gene sequence in *P*. *lactiflora* indicate that it may play an important role in cold tolerance.

Sui (2007) treated tomato plants at 4°C for different times (2, 4, 8, 12, 24, and 48 hours). Northern blot analysis revealed that tomato *GPAT* (*LeGPAT*) gene expression is induced by low temperature [40]. This analysis demonstrated that the expression of the *LeGPAT* gene initially increased and reached a peak at the fourth hour, and then gradually decreases. Chen et al. (2011) used semiquantitative RT-PCR to analyze *Lilium regale GPAT* gene expression levels at different times at 4°C [39]. The results showed that *GPAT* gene expression in *Lilium* increased after 4 hours, peaked at 16 hours, and then decreased gradually. When the mycelia from two *Volvariella volvacea* cold-resistant strains were exposed to cold stress (V23 [cold-sensitive] and VH3 [cold-tolerant]), the relative expressions of *GPAT* in the cold-tolerant strain VH3 and cold-sensitive strain V23 are different. Disparities in the expression of *GPAT* genes may result in the differential ability of the V23 and VH3 strains to respond to cold stress [41].

In our study, *PlGPAT* expression increased rapidly in *P*. *lactiflora* tissue-cultured plantlets induced with cold at 4°C and then gradually decreased. Our results are similar to the results of the two studies described above. However, the induction times to maximum *GPAT* expression between species were different, which may be due to structural differences in the specific *GPAT* genes. *PlGPAT* may be an important regulator of resistance to low-temperature stress in *P*. *lactiflora*, which is known for its hardiness to the cold. *PlGPAT* is quickly and abundantly expressed in *P*. *lactiflora* under cold stress, and *GPAT* decreased the saturated lipids in the membranes in leaf thylakoids. Thus, *Paeonia lactiflora* resists low-temperature stress. The plant adapts to the continual low temperatures gradually as enzyme expression begins to decline.

The cloning and expression analysis of *PlGPAT* represents the first step towards understanding the molecular regulation of *Paeonia* plants to cold stress. Our research on *PlGPAT* focused on the following research directions. By elucidating *PlGPAT* gene function, we were able to comprehensively understand the molecular mechanism of cold resistance in *P*. *lactiflora*. While it is not known whether the structure gene is compatible with its function, we demonstrated that the function of the *GPAT* gene may be very different across plant species. Therefore, further examination of the characteristics of the *PlGPAT* gene structure would be useful, as it would better reveal the reciprocal relation between structure and function. Finally, hardiness to the cold in plants is a quantitative trait, and this signifies that it is subject to regulation by multiple genes. This relationship requires further investigation, including focusing on both the mechanism of cold hardiness due to *PlGPAT* as well as how this gene works with other genes and transcription factors in *P*. *lactiflora* to resist cold stress. The *GPAT* gene sequences and GenBank accession numbers are provided in the Supplementary file.

## Supporting information

S1 File The list of *GPAT* gene sequences and GenBank accession numbers. (DOCX)
