# Supplementary material for: Characterization of the glycerol-3-phosphate acyltransferase gene and its real-time expression under cold stress in Paeonia lactiflora Pall
Source: PLoS One. 2018 Aug 10;13(8):e0202168. doi: 10.1371/journal.pone.0202168 (PMC6086452; doi:10.1371/journal.pone.0202168)
Supplement: S1 File — (DOCX) [file pone.0202168.s001.docx]

**S1 File. The list of *GPAT* gene sequences and GenBank accession numbers**

The *GPAT* gene sequences and GenBank accession numbers are: *Paeonia lactiflora* (AJT34990), *Vitis vinifera* (XP_002276101.1)*, Thebroma cacao* (XP_007040713.1)*, Fragaria vesca* (XP_004300201.1)*, Prunus persica* (XP_020419576), *Camellia sinensis* (AGS77295.1)*, Glycine max* (XP_006587606.1)*, Jatropha curcas* (ACR61638.1)*, Cucumis sativus* (XP_004136204.1)*, Ricinus communis* (XP_00251899 3.1)*, Malus domestica* (AHG59378.1)*, Helianthus annuus* (ADV16382.1)*, Cucurbita ficifolia* (AAD11604.1)*, Citrus unshiu* (BAB79529.1)*, Solanum tuberosum* (XP_00635289 8.1)*, Elaeis guineensis* (AAF64066.1)*, Zea mays* (NP_001152109.1)*, Oryza sativa* ssp. *japonica* (AAP55165)*, Capsicum annuum* (AAP79 443.2)*,* and *Sorghum bicolor* (XP_002467426.1), *Amborella trichopoda* (XP_006854076.1), *Lupinus angustifolius* (XP_019423896), *Nicotiana tabacum* (XP_016475022), *Sesamum indicum* (XP_011069706), *Populus euphratica* (XP_011018484), *Gossypium hirsutum* (XP_016717835.1), *Cephalotus follicularis* (GAV84421.1), *Ziziphus jujuba* (XP_015875079.1), *Erythranthe guttata* (XP_012843053.1), *Ipomoea nil* (XP_019190862.1), *Cajanus cajan* (XP_020216491.1).
